# A Novel Ferroptosis-Related Gene Signature to Predict Prognosis in Patients with Head and Neck Squamous Cell Carcinoma

**DOI:** 10.1155/2021/5759927

**Published:** 2021-11-22

**Authors:** Li Xu, Ying-ying Li, Yang-chun Zhang, Yong-xu Wu, Dan-dan Guo, Dan Long, Zhao-hui Liu

**Affiliations:** Department of Otorhinolaryngology, Head and Neck Surgery, Affiliated Hospital of Zunyi Medical University, Zunyi, 563000 Guizhou Province, China

## Abstract

The clinical TNM staging system is currently used to evaluate the prognosis of head and neck squamous cell carcinoma (HNSCC). The 5-year survival rate for patients with HNSCC is less than 50%, which is attributed to the lack of reliable prognostic biomarkers. Ferroptosis-related genes (FRGs) regulate cancer initiation and progression. Therefore, we analyzed the correlation between FRGs and the clinical outcomes of patients with *HNSCC*. A typical prognostic model of FRGs for HNSCC was constructed using bioinformatics tools and data from public databases, including The Cancer Genome Atlas (TCGA), Gene Expression Omnibus (GEO), and GeneCards. The model was generated based on the following six FRGs: *ATG5*, *PRDX6*, *OTUB1*, *FTH1*, *SOCS1*, and *MAP3K5*. The accuracy of model prediction was analyzed systematically. The overall survival (OS) of the high-risk group was significantly lower than that of the low-risk group. The AUC for 1-year, 3-year, and 5-year survival were 0.645, 0.721, and 0.737, respectively, in the training set (TCGA cohort) and 0.726, 0.620, and 0.584, respectively, in the validation set (GSE65858). The multivariate Cox regression analysis revealed that the risk score was an independent prognostic factor for HNSCC. Gene Ontology (GO) and Kyoto Encyclopedia of Genes and Genomes (KEGG) analyses revealed that six FRGs were enriched in the ferroptosis pathway. A novel FRG prognostic signature model was established for HNSCC. The findings of this study reveal that FRGs are potential biomarkers for HNSCC.

## 1. Introduction

Head and neck carcinoma (HNC), which is one of the top 10 tumors, accounts for 3% of all cancer cases. Each year, 900,000 new HNC cases and 500,000 HNC-related deaths are reported [1]. Head and neck squamous cell carcinoma (HNSCC) is the most common histological subtype of head and neck tumors [2]. Risk factors for HNSCC include smoking, drinking, and human papillomavirus (HPV) infection [3]. The current method for confirming the diagnosis of HNSCC remains pathologic histological examination [4]. Due to lack of indicators for early diagnosis, HNSCC is not easily detected and diagnosed, and approximately 60% of patients with HNSCC are at an advanced stage by the time they receive treatment [5]. The main treatment methods for focal or locally limited HNSCC are resection, radiotherapy, and systemic therapy [4]. Currently, the clinical TNM staging system is used to evaluate HNSCC prognosis. However, the predictive effect is not satisfactory, and the survival rate of patients with HNSCC is less than 50% [6]. With the advent of precision medicine, patients need personalized treatment, and studies of drug treatments targeting biomarkers have shown good results [7].

The genetic, biochemical, and morphological characteristics of ferroptosis are classified under the category of iron-dependent cell death and accumulation of superoxide lipids [8]. Ferroptosis has both advantages and disadvantages because it can promote tumor progression by upregulating DNA replication or inhibit tumor progression by enhancing cell death [9–11]. The induction of iron death has emerged as a promising therapeutic approach for triggering cancer cell death, particularly in malignancies that are resistant to conventional treatment [12, 13]. Few studies have been conducted on iron-induced death in head and neck cancers, and even fewer studies have suggested that ferroptosis is associated with HNSCC pathogenesis [14, 15]. Therefore, there is an urgent need to identify reliable biomarkers of ferroptosis.

The tumor microenvironment (TME) plays an important role in modern cancer treatment [16]. Tumor-infiltrating immune cells (TICs) are indispensable components of the TME [17]. In particular, the composition and activity of TICs at the tumor site are considered prognostic factors for many cancers [18]. The TME of HNSCC contains an increased number of immune infiltrates. The clinical efficacy of cancer immunotherapy against HNSCC has been demonstrated recently [19]. Previous studies reported that iron metabolism markedly influenced the TME and that the iron requirements of cancer cells were higher than those of nontumorous cells [20]. Therefore, the prognostic value of FRG in HNSCC must be evaluated.

In this study, correlations between the expression profiles of FRGs and clinical outcomes in patients with HNSCC were analyzed using clinical datasets curated in public databases. The Cox regression analysis was performed to analyze the prognostic value of six FRGs in HNSCC. Additionally, the correlation between these six FRGs and the TME of HNSCC was examined. The findings of this study reveal that FRGs are potential biomarkers for HNSCC.

## 2. Materials and Methods

### 2.1. Data Acquisition

RNA sequence and clinical information of 494 HNSCC cases were collected from TCGA database using the R package “TCGAbiolinks.” A total of 44 normal samples and 8 samples with missing survival data were excluded. A validation dataset (GSE65858) containing information on 270 patients with HNSCC was downloaded from the GEO website (https://www.ncbi.nlm.nih.gov/geo/query/acc.cgi?acc=GSE65858) [21]. Four cases with missing tumor site information were excluded from the analysis; hence, only 266 patients with complete clinical data were included in our study. The keyword “ferroptosis” was used to identify 103 genes and download their data (S1) from the GeneCards database (http://www.genecards.org/) [22]. Since all data in our study were collected from public online databases, approval from the Ethics Committee or written informed consent from contributors was not required.

### 2.2. Establishing the Prognostic Signature of FRGs

The 96 FRGs in HNSCC (S2) were identified from the intersection of the 103 FRGs from the GeneCards database and all genes from TCGA dataset, and the expression data of the 96 FRGs were subsequently combined with clinical data from 494 HNSCC patients. The univariate Cox regression analysis identified eight genes associated with survival of patients with HNSCC. Meanwhile, the multivariate Cox regression model identified six prognostic ferroptosis-related genes. The genes associated with the risk of developing HNSCC were determined based on hazard ratios (HRs) as follows: HR > 1, risk-associated genes; HR < 1, protective genes. Six FRGs were evaluated using a linear combination of the Cox regression coefficients (*β*). The best model was chosen based on the lowest Akaike information criterion (AIC) value. The risk score for all patients was calculated using Equation (1):

(1) $$\begin{array}{c} \text{RiskScore}=\overset{n}{\underset{i=1}{\sum }}\text{C}\text{oef}\left(i\right)\times x\left(i\right), \end{array}$$

where Coef (*i*) represents the estimated coefficient of each FRG and *x* (*i*) represents the expression of each FRG.

### 2.3. Evaluation of the Prognostic Model

All patients in the training cohort (TCGA cohort) were divided into high-risk and low-risk groups based on the cut-off values of median risk scores. Overall survival (OS) was evaluated using the Kaplan-Meier survival curves and compared using the log-rank test. The risk curve and scatter plot of the risk score were generated using the R software package “pheatmap.” Principal component analysis (PCA) was performed to visualize sample distribution. The R package “timeROC” was used to generate time-dependent receiver operating characteristic (ROC) curves, and area under the curve (AUC) plots were generated for the 1-year, 3-year, and 5-year survival rates to assess the predictive performance of the risk scoring model. Univariate and multivariate Cox regression analyses were used to assess the applicability of the prognostic model independent of other clinicopathological factors of patients with HNSCC, including age, sex, grade, clinical stage, T-stage, and risk score. N-stage and M-stage were not analyzed because these data were missing for some of the patients. To assess the net benefit to patients, we used the R package “stdca.R” for decision curve analysis (DCA) and plotted DCA curves for 1 year, 3 years, and 5 years. Finally, we validated model accuracy using the validation cohort (GSE65858).

### 2.4. Stratified Analyses of the Expression Levels of Six FRGs

Differential expression of six ferroptosis-related genes was analyzed between high-risk and low-risk groups from TCGA cohort using the “ggpubr” R package. To explore the clinical significance of these six ferroptosis-related genes, the patients were stratified according to clinical parameters (age, sex, grade, clinical stage, T-stage, N-stage, HPV-in situ hybridization (ISH) results, and P16 status). Only significant results are given in this study. The clinical values of sex and M-stage are not shown.

### 2.5. Gene Ontology (GO) Terms, Kyoto Encyclopedia of Genes and Genomes (KEGG) Pathways, and Protein-Protein Interaction (PPI) Network

The six FRGs were subjected to GO and KEGG enrichment analyses using the R package “clusterProfiler” and “http://org.Hs.eg.db/”. *P* values were adjusted with the “BH” method. The PPI network was constructed using STRING version 11.0 (https://string-db.org/) [23].

### 2.6. CIBERSORT Analysis

CIBERSORT [24] was used to calculate the relative proportions of 22 TICs in each HNSCC sample. Spearman's correlation analysis was used to analyze the association between TICs and the six FRGs. Only significant (*P* < 0.001) results are shown.

### 2.7. Statistical Analysis

Data extraction, cleaning, and merging were performed using the R packages “tidyverse” and “stringr.” The R package “Tableone” was used to tabulate and analyze the baseline data. All Cox regressions were conducted using the R “survival” package. Overall survival between the subgroups was compared using the log-rank test. Differential gene expression between different subgroups was analyzed using the Wilcoxon test. The R packages “ggplot2,” “forestplot,” “cowplot,” “survminer,” “timeROC,” and “ggpubr” were used for visualization. All statistical analyses and visualizations were performed using the R software version 3.6.3 (https://www.r-project.org/). Differences were considered significant at *P* < 0.05, unless otherwise stated. All *P* values were calculated using two-sided tests (^∗^*P* < 0.05, ^∗∗^*P* < 0.01, ^∗∗∗^*P* < 0.001, ^∗∗∗∗^*P* < 0.0001).

## 3. Results

### 3.1. Study Design (Figure 1)

A total of 494 patients with HNSCC from TCGA cohort and 266 patients with HNSCC from the GSE65858 dataset were included. Detailed clinical information of the patients is summarized in Table 1.

### 3.2. Identification of Prognostic FRGs in TCGA Cohort

**Figure 2:**
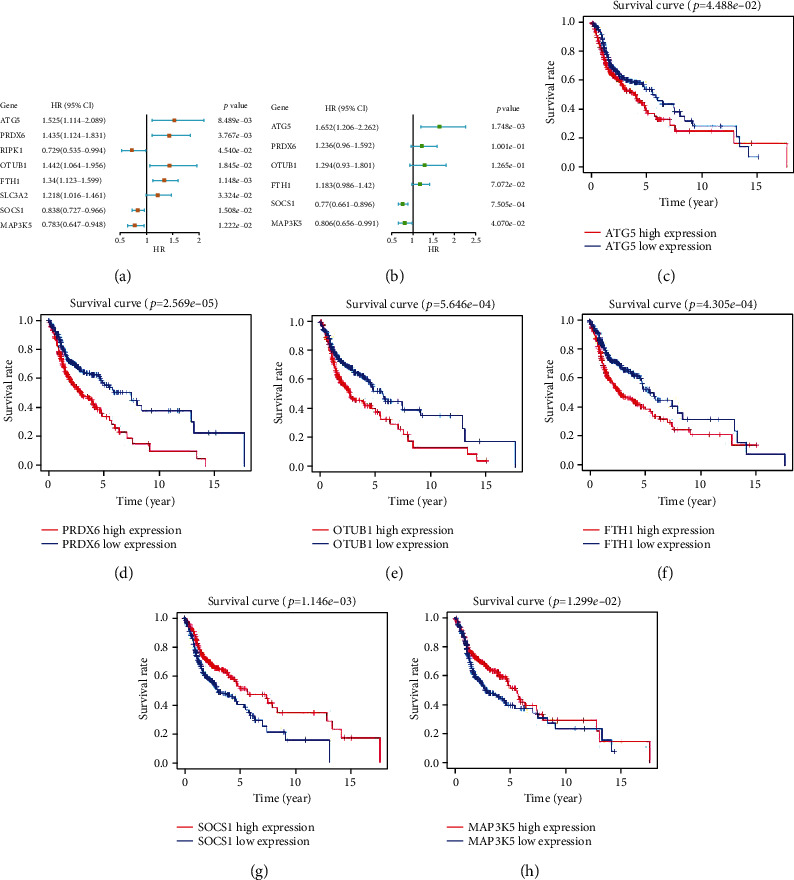
Construction of ferroptosis-associated signature gene model. (a) Forest map of eight ferroptosis-associated genes correlated with the survival of patients with HNSCC was obtained using the univariate Cox regression analysis. (b) Forest maps of six ferroptosis-related genes (FRGs) were generated using the multivariate Cox regression analysis. (c–h) Kaplan-Meier survival curves of the high-expression and low-expression groups of six FRGs.

Based on the expression levels of 96 FRGs, the univariate Cox regression analysis revealed that eight FRGs were associated with OS (Figure 2(a)). A prognostic model was constructed for six FRGs based on the expression levels of eight FRGs and the multivariate Cox regression analysis. The best prognostic gene signature was selected based on the lowest AIC value (Table 2). Among them, *ATG5*, *PRDX6*, *OTUB1*, and *FTH1* were classified as risk-associated genes (HR > 1), whereas *SOCS1* and *MAP3K5* were classified as protective genes (HR < 1) (Figure 2(b)). Kaplan-Meier survival curves were plotted based on expression levels (high-expression and low-expression) of six FRGs. The OS of patients with HNSCC in the *ATG5*, *PRDX6*, *OTUB1*, and *FTH1* high-expression groups was lower than that of patients in the low-expression groups (Figures 2(c)–2(f)). Conversely, the OS of patients with HNSCC in the *SOCS1* and *MAP3K5* low-expression groups was lower than that of patients in the high-expression groups (Figures 2(g) and 2(h)).

### 3.3. Evaluation of Prognostic Models

**Figure 3:**
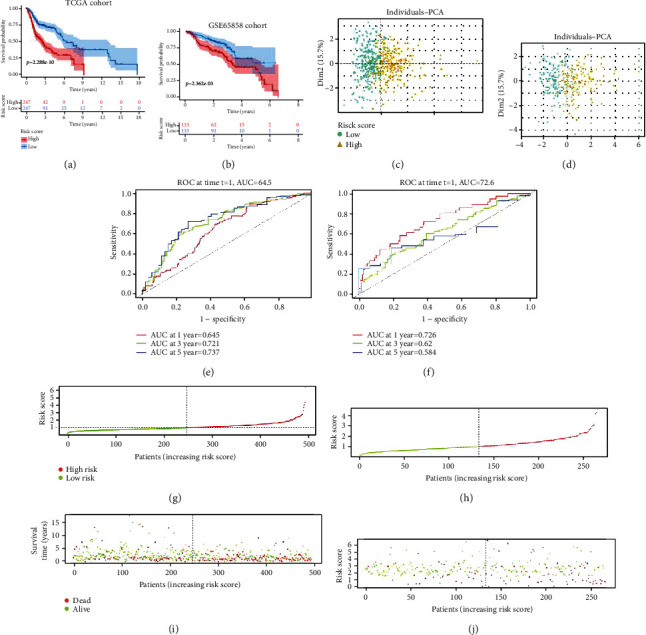
Validation of the prognostic model. (a, b) The Kaplan-Meier survival analysis of the high-risk and low-risk groups in The Cancer Genome Atlas (TCGA) and GSE65858 datasets. (c, d) Principal component analysis of the high-score and low-score groups in TCGA and GSE65858 datasets. (e, f) The area under the curve values for the 1-year, 3-year, and 5-year survival rates of TCGA and GSE65858 datasets. (g, h) The risk score distribution curve of high-risk and low-risk groups in TCGA and GSE65858 datasets. (i, j) Scatter plot indicating the association between survival time and risk score in TCGA and GSE65858 datasets.

**Figure 4:**
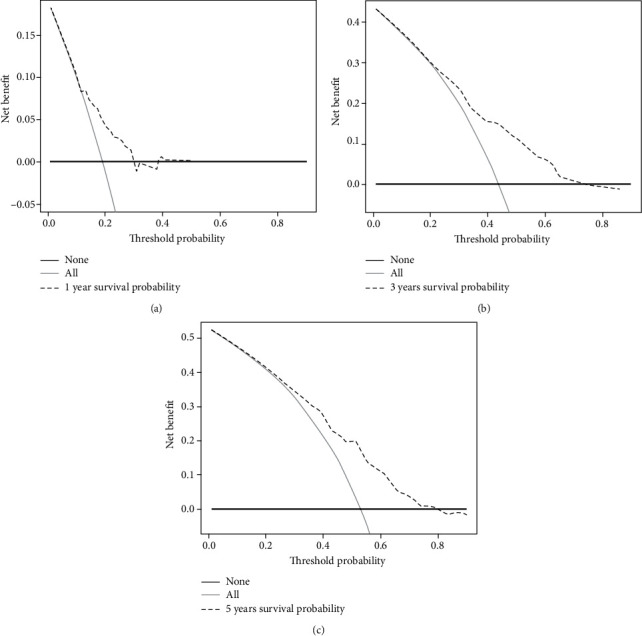
The decision curve analysis (DCA) results showed a benefit in 1-year (a), 3-year (b), and 5-year (c) survival for patients in this prediction model.

A prognostic model was constructed to compute the hazard score for each HNSCC patient in TCGA dataset using the following equation: risk score = [(0.5019 × *ATG*5 expression) + (−0.2152 × *MAP*3*K*5 expression) + (−0.2619 × *SOCS*1 expression) + (0.2577 × *OTUB*1 expression) + (0.1684 × *FTH*1 expression) + (0.2121 × *PRDX*6 expression)]. Based on the median risk score (0.960), all patients were divided into two groups: high-risk group (*n* = 247) and low-risk group (*n* = 247). The Kaplan-Meier survival curve showed that the OS of the high-risk group was significantly lower than that of the low-risk group (Figure 3(a)). The 1-year, 3-year, and 5-year OS rates of the high-risk group were 74.60%, 40%, and 29.30%, respectively, while those of the low-risk group were 89.10%, 71.90%, and 61.10%, respectively. PCA was performed to examine the significant risk score distribution differences between the low-risk and high-risk groups (Figure 3(c)).  Figure 3(e) shows the AUC values for the 1-year (0.645), 3-year (0.721), and 5-year (0.737) survival rates. The horizontal ordinate axis of the risk score curve and survival events were sorted according to the risk score (Figure 3(g)). Patients with high-risk scores exhibited decreased survival and increased death rates (Figure 3(i)). The prognostic model was validated using the GSE65858 cohort (*n* = 266). The OS of the high-risk group (*n* = 133) was significantly lower than that of the low-risk group (*n* = 133) (Figure 3(b)). The 1-year, 3-year, and 5-year OS rates of the high-risk group were 78.95%, 54.33%, and 36.81%, respectively, while those of the low-risk groups were 92.5%, 72%, and 51.50%, respectively. PCA was performed to demonstrate the significant risk score distribution differences between the low-risk and high-risk groups in the validation cohort (Figure 3(d)).  Figure 3(f) shows the AUC values for the 1-year (0.726), 3-year (0.62), and 5-year (0.584) survival rates. The horizontal ordinate axis of the risk score curve and survival event data were sorted according to the risk scores (Figure 3(h)). Patients with high-risk scores exhibited decreased survival rates and increased mortality rates (Figure 3(j)). The DCA results showed a benefit in 1-year, 3-year, and 5-year survival for patients in this prediction model (Figures 4(a)–4(c)).

### 3.4. Risk Score Is an Independent Prognostic Factor

**Figure 5:**
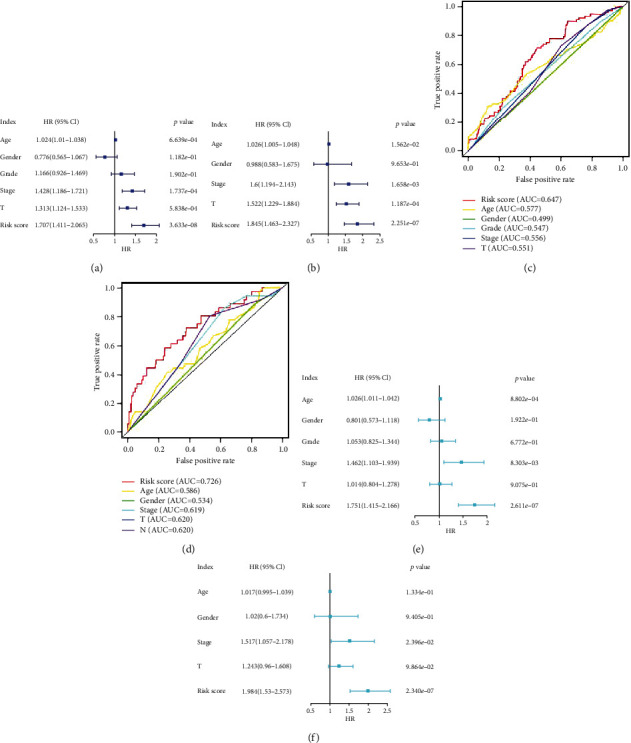
Independent prognostic factor analysis. Results of the univariate Cox regression analysis to determine the association between overall survival rate and clinical characteristics in The Cancer Genome Atlas (TCGA) cohort (a) and GSE65858 datasets (b). The receiving operating characteristic curve was generated based on the risk score and clinical characteristics of TCGA (c) and GSE65858 (d) datasets. Results of the multivariate Cox regression analyses to determine the association between overall survival rate and clinical characteristics in TCGA (e) and GSE65858 (f) datasets.

In TCGA cohort (training set), the univariate Cox regression analysis revealed that age (*P* = 6.639*e* − 04), tumor stage (*P* = 1.737*e* − 04), T-stage (*P* = 5.838*e* − 04), and risk score (*P* = 3.633*e* − 08) were significantly correlated with OS (Figure 5(a)), while the multivariate Cox regression analysis revealed that age (*P* = 8.802*e* − 04), tumor stage (*P* = 8.303*e* − 03), and risk score (*P* = 2.611*e* − 07) were significantly correlated with OS (Figure 5(e)). As shown in Figure 5(c), the AUC for the risk score (0.647) was higher than that for age (0.577), gender (0.499), tumor grade (0.547), tumor stage (0.556), and T-stage (0.551). The univariate Cox analysis of the GSE65858 cohort (validation set) revealed that age (*P* = 1.562*e* − 02), tumor stage (*P* = 1.658*e* − 03), T-stage (*P* = 1.187*e* − 04), and risk score (*P* = 2.251*e* − 07) were significantly correlated with OS (Figure 5(b)). Meanwhile, the multivariate Cox analysis revealed that tumor stage (*P* = 2.396*e* − 02) and risk score (*P* = 2.340*e* − 07) were significantly associated with OS (Figure 5(f)). As shown in Figure 5(d), the AUC for the risk score (0.726) was higher than that for age (0.586), sex (0.534), tumor stage (0.619), T-stage (0.620), and N-stage (0.620). These results suggest that FRGs are independent prognostic factors for HNSCC.

### 3.5. Stratified Analysis of Six FRGs Based on Clinical Characteristics

**Figure 6:**
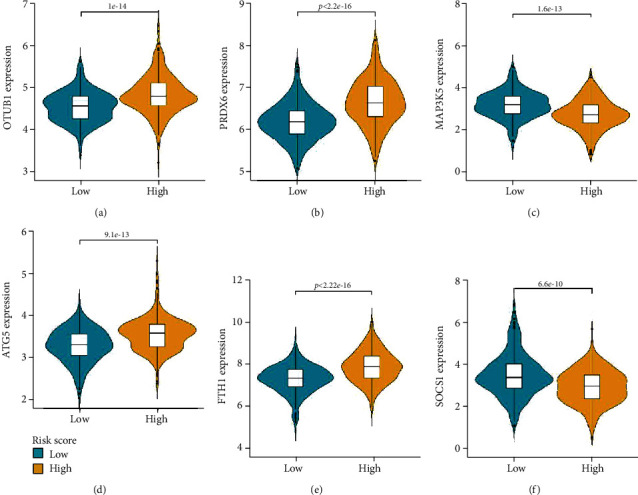
Differential expression of six ferroptosis-related signature genes. (a–f) The expression levels of *OTUB1*, *PRDX6*, *MAP3K5*, *ATG5*, *FTH1*, and *SOCS1* in the high-risk score and low-risk score groups.

**Figure 7:**
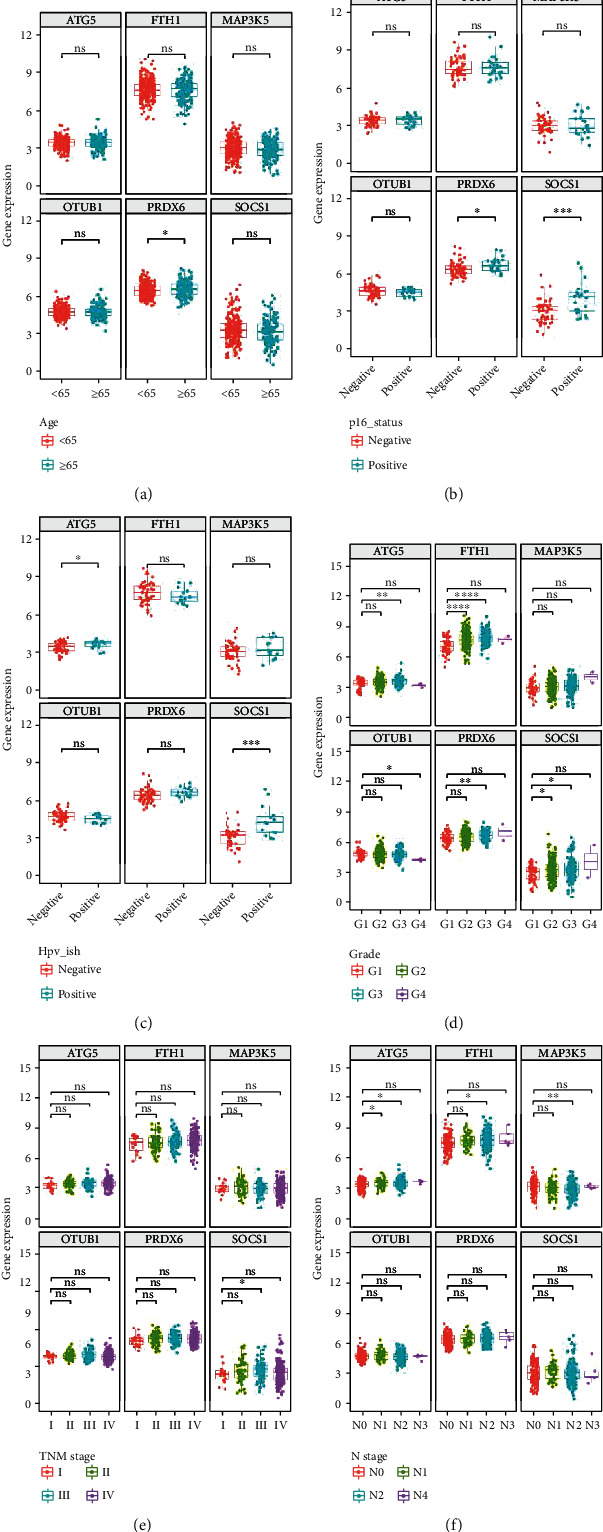
Expression levels of six ferroptosis-related signature genes based on the age (a), P16 status (b), human papillomavirus-in situ hybridization result (c), tumor grade (d), tumor stage (e), and T-stage (f) of patients in The Cancer Genome Atlas (TCGA) cohort.

The six FRGs were differentially expressed between the high-risk and low-risk groups. The expression of *ATG5*, *PRDX6*, *OTUB1*, and *FTH1* was upregulated in the high-risk group (Figures 6(a), 6(b), 6(d), and 6(e)), whereas that of *SOCS1* and *MAP3K5* was downregulated in the high-risk group (Figures 6(c) and 6(f)). The correlations between the six FRGs and clinical parameters such as age, P16 status, HPV-ISH result, tumor grade, TNM stage, and N-stage were analyzed. The expression levels of *PRDX6* in patients aged ≥ 65 years were higher than those in patients aged < 65 years (Figure 7(a)). Compared with those in the P16-positive group, the expression levels of *SOCS1* and *PRDX6* were upregulated in the P16-negative groups (Figure 7(b)). Additionally, the expression levels of *SOCS1* and *ATG5* in the HPV-ISH-positive group were higher than those in the HPV-ISH-negative group (Figure 7(c)). The expression level of *ATG5* varied between G1 and G3 grades, while that of *FTH1* varied between G1 and G2 grades, as well as between G1 and G3 grades. *OTUB1* was differentially expressed between G1 and G4 grades, whereas *PRDX6* was differentially expressed between G1 and G3 grades. The expression level of *SOCS1* varied between G1 and G2 grades, as well as between G1 and G3 grades (Figure 7(d)). The expression level of *SOCS1* in stage III tumors was higher than in stage I tumors (Figure 7(e)). The expression level of *ATG5* varied between N0 and N1 stages, as well as between N0 and N2 stages. *FTH1* and *MAP3K5* were differentially expressed between N0 and N2 stages (Figure 7(f)).

### 3.6. GO, KEGG, and PPI Analyses

**Figure 8:**
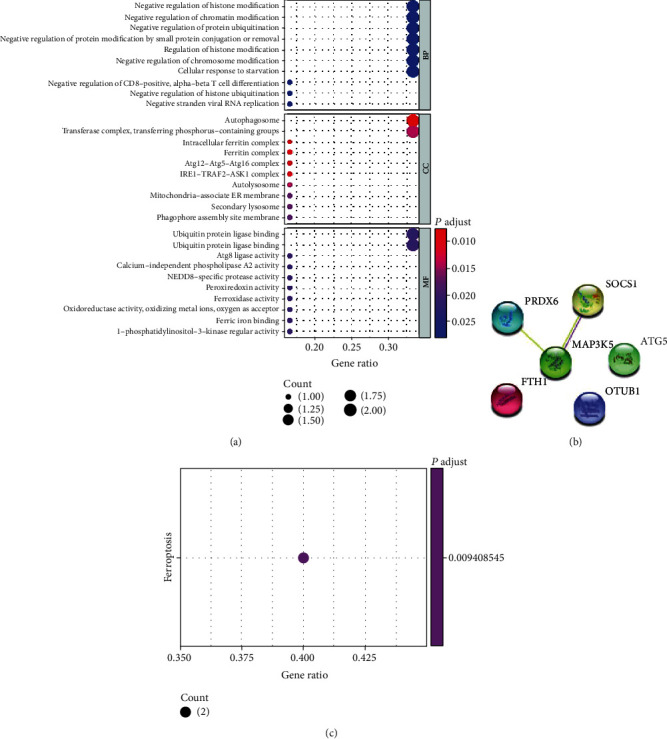
Biological function analysis of six ferroptosis-related signature genes. (a) Gene Ontology analysis. (b) Construction of protein-protein interaction network. (c) Kyoto Encyclopedia of Genes and Genomes analysis.

The top two GO results for biological processes were negative regulation of histone modification and negative regulation of chromatin organization. The top two GO results for cellular components were autophagosomes, transferase complex, and transferring phosphorus-containing groups. The top two GO results for molecular functions were ubiquitin protein ligase binding and ubiquitin-like protein ligase binding (Figure 8(a)). The KEGG analysis revealed that ferroptosis was the most significantly enriched pathway (Figure 8(c)). The PPI network revealed correlations between *MAP3K5*, *SOCS1*, and *PRDX6*. *FTH1*, *OTUB1*, and *ATG5* did not form part of the network (Figure 8(b)).

### 3.7. Correlation between Ferroptosis and TIC Infiltration in TCGA Cohort

**Figure 9:**
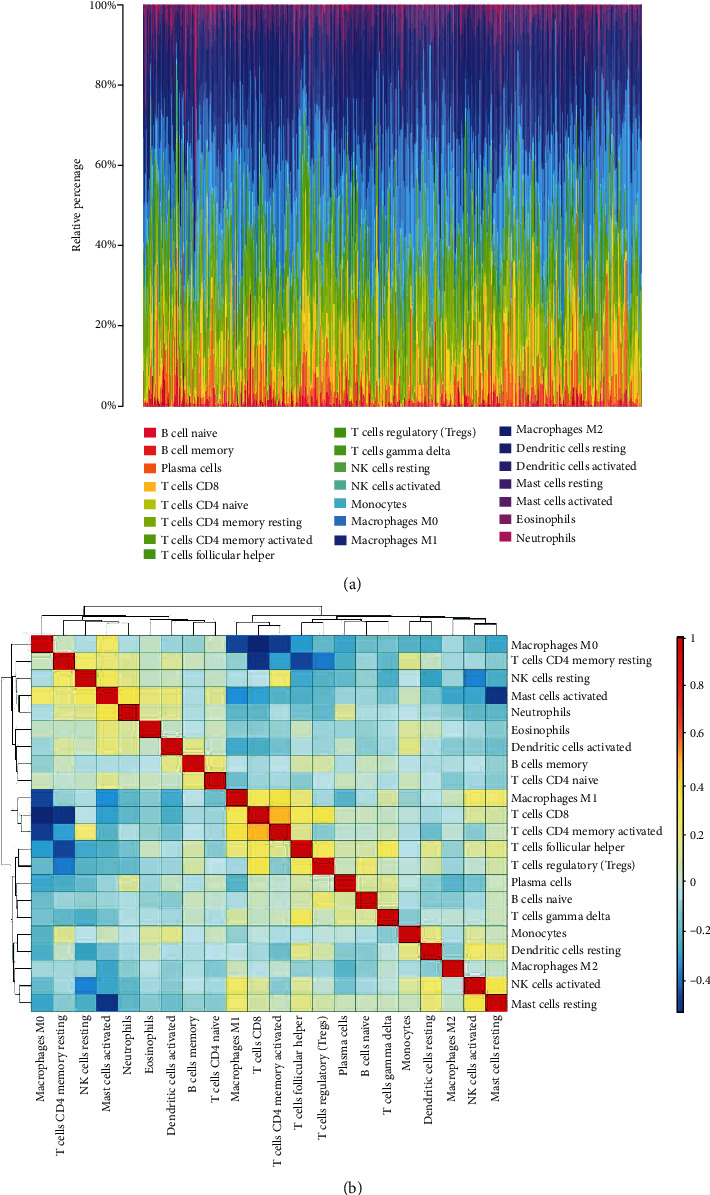
CIBERSORT analysis of The Cancer Genome Atlas (TCGA) cohort. (a) Histogram of relative proportions of 22 tumor immune cells (TICs). (b) Correlation among 22 tumor immune cells (TICs).

**Figure 10:**
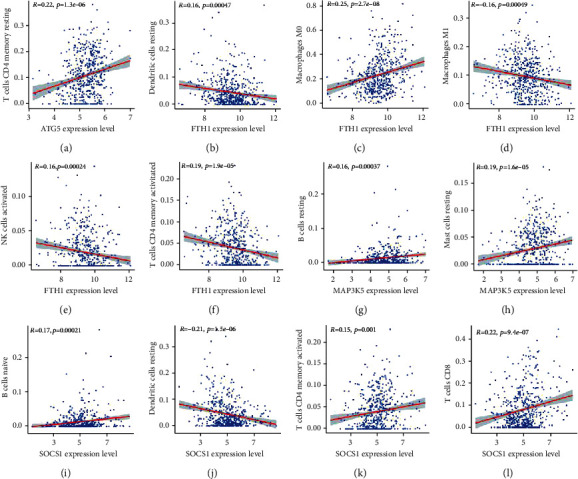
Association between the distribution of tumor immune cells and expression of ferroptosis-related genes (FRGs) in head neck squamous cell carcinoma. (a) ATG5 was positively associated with the proportion of CD4 resting memory T cells. (c) FTH1 was positively associated with the proportion of M0 macrophages. (b, d–f) FTH1 was negatively correlated with the proportion of resting dendritic cells, M1 macrophages, activated natural killer cells, and activated CD4 memory T cells. (g, h) MAP3K5 was positively associated with the proportion of naive B cells and resting mast cells. (i–l) SOCS1 was positively correlated with the proportion of naive B cells, activated CD4 memory T cells, and CD8 T cells but negatively correlated with the proportion of resting dendritic cells.

To investigate the correlation between ferroptosis and TIC infiltration, the “CIBERSORT” algorithm was used to analyze the relative proportion of immune cells in the top 22 HNSCC samples (Figure 9(a)). The association between the 22 TIC proportions and ferroptosis was represented using a heatmap (Figure 9(b)). A scatter plot showing the association between the expression of FRGs and the proportion of 22 TICs in HNSCC samples revealed a positive association between ATG5 and resting CD4 memory T cells (Figure 10(a)). Additionally, resting dendritic cells, M1 macrophages, activated natural killer cells, and activated CD4 memory T cells were negatively correlated with FTH1, whereas macrophage M0 was positively correlated with FTH1 (Figures 10(b)–10(f)). Naive B cells and resting mast cells were positively correlated with MAP3K5 expression (Figures 10(g) and 10(h)). Furthermore, naive B cells, activated CD4 memory T cells, and CD8 T cells were positively correlated with SOCS1, whereas resting dendritic cells were negatively correlated with SOCS1 (Figures 10(i)–10(l)).

## 4. Discussion

The incidence of HNSCC is increasing worldwide [1]. The 5-year survival rate of patients with HNSCC is less than 50% [6], which is attributed to lack of reliable prognostic biomarkers [25]. Recent studies have established a correlation between molecular markers such as autophagy genes, immune genes, autophagy-associated long noncoding RNAs (lncRNAs), and immune-related lncRNAs, and HNSCC prognosis [26–29], which may aid in determining clinical outcomes.

As ferroptosis is reportedly involved in both cancer progression and cancer suppression [30], it can be a novel therapeutic target for tumors. As such, prognostic ferroptosis-related signature genes have been established for various tumors, including hepatocellular carcinoma [31], glioma [32], uveal melanoma [33], and clear cell renal cancer [34]. Low concentrations of PTX and RSL3 synergistically suppress hypopharyngeal squamous carcinoma by inducing ferroptosis [15]. SLC7A11 is a biomarker and therapeutic target for HPV-positive HNSCC [14]. Additionally, ferroptosis enhances the clinical growth-inhibitory efficacy of PDT against oral tongue squamous cell carcinoma [35]. Previous studies have mainly focused on HNSCC pathogenesis, as well as on the effects of drugs and surgical treatment of HNSCC. However, the correlation between ferroptosis and HNSCC prognosis has not previously been examined. This study demonstrated that FRGs are prognostic markers of HNSCC. In addition to the internal validation of TCGA dataset, this study validated the prognostic value of FRGs using the GSE65858 dataset. The findings of this study indicate that FRGs can predict the OS of patients with HNSCC.

Several studies have also suggested a close correlation between ferroptosis and tumor immunotherapy. Ferroptosis plays a critical role in the efficacy of tumor immunotherapy [36]. In cancer immunotherapy, CD8(+) T cells exert antitumor effects by promoting tumor ferroptosis [37]. Additionally, a chemically programmed vaccine targeting ferroptosis and immunity has been developed [38]. Thus, the ferroptosis pathway is a potential therapeutic target for tumors. However, further studies are needed to examine the role of ferroptosis in the immune environment of cancer. In this study, TCGA dataset was analyzed using the CIBERSORT algorithm to demonstrate the correlation between 22 TICs and FRGs.

Six ferroptosis-related signature genes were subjected to GO and KEGG analyses, and a PPI network was constructed. The correlation between these genes and 22 TICs in HNSCC was examined. SOCS1 has been identified as an immune-related prognostic protective gene for HNSCC [39]. One study in an Indian population suggested that SOCS1 may mediate immunosuppression in HPV-positive tumors [40]. Treatment with resveratrol suppressed HNSCC proliferation through SOCS1 [41], suggesting that SOCS1 is a potential therapeutic target for HNSCC. This is in agreement with the findings of the present investigation, which demonstrated that the expression of SOCS1 was upregulated in HPV-positive patients in both the P16-positive and HPV-ISH-positive groups in TCGA cohort. The expression of SOCS1 was positively correlated with the proportion of naive B cells, activated CD4 T cells, and CD8 T cells. In contrast, the expression of SOCS1 was negatively correlated with the proportion of resting dendritic cells. These findings demonstrate the role of SOCS1 in HNSCC.

ATG5 has previously been reported to be associated with autophagy. Recent studies have reported that ATG5 regulates the migration, invasion, and apoptosis of HNSCC through autophagy [42, 43]. Additionally, ATG5 determines the sensitivity of HNSCC to radiotherapy and chemotherapy [44–47]. The expression of ATG5 was upregulated in the HPV-positive group, indicating that it regulates radiotherapy sensitivity of HPV-positive tumors. GO analysis revealed that ATG5 was enriched in cellular components containing autophagosomes. The expression of ATG5 was upregulated in advanced-grade and N-stage tumors.

Radiotherapy exerts growth-inhibitory effects against HNSCC by promoting redox sensitivity through MAP3K5 [48, 49]. The expression of MAP3K5 was downregulated in advanced N-stage tumors, which is consistent with the downregulated expression of MAP3K5 in the high-risk score group and increased survival rate in the MAP3K5 high-expression group. MAP3K5 was positively correlated with the proportion of naive B cells and resting mast cells.

PRDX6 suppresses apoptosis in HNSCC by exerting antioxidant effects [50]. The expression of PRDX6 was upregulated in the high-risk score group. Additionally, the PRDX6 high-expression group exhibited decreased OS. Furthermore, PRDX6 expression was upregulated in the subgroup with poor histopathological differentiation. These results suggest that PRDX6 is an oncogenic factor in HNSCC.

The correlation between HNSCC, OTUB1, and FTH1 has not previously been reported. OTUB1 is involved in ubiquitination in breast cancer [51]. GO enrichment analysis of six FRGs revealed that the terms biological processes and molecular functions also include ubiquitin-related mechanisms. Survival analysis revealed that the OTUB1 high-expression group exhibited low OS and that the expression of OTUB1 was upregulated in the high-risk score group. The results of this study suggest that OTUB1 is a risk factor for HNSCC, although further experimental studies are needed.

The National Center for Biotechnology Information database classifies FTH1 as a pseudogene. However, the role of FTH1 in tumor development has recently been reported. FTH1 functions as a neoplastic suppressor in non-small cell lung cancer [52], breast cancer [53], and ovarian cancer [54]. However, FTH1 functions as a tumor suppressor in metastatic melanoma [55]. In this study, FTH1 was identified as a risk factor for HNSCC. The OS of the FTH1 high-expression group was low. Additionally, the expression of FTH1 was upregulated in the high-risk score group.

This study had some limitations. This was a retrospective study and established gene signatures based on data from public databases. Thus, the gene signature requires further validation in prospective studies and multicenter clinical trials. Additionally, the mechanisms underlying the association between ferroptosis-related genes and tumor immunity in HNSCC remain poorly understood and warrant further investigation.

## 5. Conclusions

This study identified and validated a clinical prognostic model based on six FRGs, which served as independent prognostic factors for patients with HNSCC. These genes may also serve as potential prognostic biomarkers for HNSCC.

## Figures and Tables

**Figure 1:**
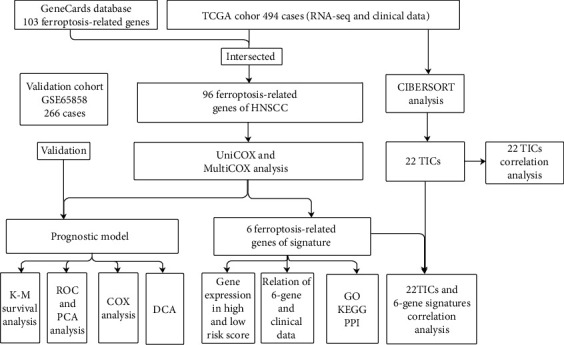
Flow chart of the study plan. TCGA: The Cancer Genome Atlas; TICs: tumor-infiltrating immune cells; K-M: Kaplan-Meier; ROC: receiving operating characteristic; PCA: principal coordinate analysis; GO: Gene Ontology; KEGG: Kyoto Encyclopedia of Genes and Genomes; PPI: protein-protein interaction; HNSCC: head and neck squamous cell carcinoma, DCA: decision curve analysis.

**Table 1:** Baseline clinical characteristics of patients with HNSCC in TCGA and GSE65858 cohorts.

Clinical characteristics	TCGA		GSE65858	
	Total (*n* = 494)	%	Total (*n* = 266)	%
*Age*				
<65	306	61.9	180	67.7
≥65	188	38.1	86	32.3
*Gender*				
Female	132	26.7	45	16.9
Male	362	73.3	221	83.1
*Grade*				
G1	61	12.3	—	—
G2	294	59.5	—	—
G3	118	23.9	—	—
G4	2	0.4	—	—
GX	16	3.2	—	—
Unknown	3	0.6	—	—
*Stage*				
I	24	4.9	18	6.8
II	72	14.6	37	13.9
III	78	15.8	36	13.5
IV	256	51.8	175	65.8
Unknown	64	13.0	—	—
*T-stage*				
T0	1	0.2	—	—
T1	44	8.9	35	13.2
T2	131	26.5	80	30.1
T3	95	19.2	56	21.1
T4	169	34.2	95	35.7
TX	32	6.5	—	—
Unknown	22	4.5	—	—
*N-stage*				
N0	168	34.0	93	35.0
N1	65	13.2	31	11.7
N2	162	32.8	130	48.9
N3	7	1.4	12	4.5
NX	68	13.8	—	—
Unknown	24	4.9	—	—
*M-stage*				
M0	181	36.6	259	97.4
M1	1	0.2	7	2.6
MX	61	12.3	—	—
Unknown	251	50.8	—	—
*HPV status*				
HPV16	—	—	60	22.6
Other HPV	—	—	13	4.9
Negative	—	—	192	72.2
Unknown	—	—	1	0.4
*HPV16*				
DNA(+) RNA(+)	—	—	35	13.2
DNA(+) RNA(-)	—	—	19	7.1
DNA(-)	—	—	192	72.2
Unknown	—	—	20	7.5
*P16*				
Negative	72	14.6	—	—
Positive	30	6.1	—	—
Unknown	392	79.4	—	—
*HPV-ISH result*				
Negative	64	13.0	—	—
Positive	19	3.8	—	—
Unknown	411	83.2	—	—

HNSCC: head and neck squamous cell carcinoma; HPV: human papillomavirus; TCGA: The Cancer Genome Atlas; ISH: in situ hybridization.

**Table 2:** Akaike information criterion (AIC) of the models.

Model	Prognostic signature combination	AIC
1	*ATG5 + PRDX6 + RIPK1 + OTUB1 + FTH1 + SLC3A2 + SOCS1 + MAP3K5*	2289.81
2	*ATG5 + PRDX6 + RIPK1 + OTUB1 + FTH1 + SOCS1 + MAP3K5*	2287.82
3	*ATG5 + PRDX6 + OTUB1 + FTH1 + SOCS1 + MAP3K5*	2285.87

## Data Availability

The data that support the findings of this study are available from the corresponding author upon reasonable request.

## Supplementary Materials

Supplementary Materials See S1 and S2 datasets.
