# Supplementary material for: A Novel Ferroptosis-Related Gene Signature to Predict Prognosis in Patients with Head and Neck Squamous Cell Carcinoma
Source: Dis Markers. 2021 Nov 22;2021:5759927. doi: 10.1155/2021/5759927 (PMC8629675; doi:10.1155/2021/5759927)
Supplement: Supplementary Materials — See S1 and S2 datasets. [file 5759927.f1.zip › 5759927.f1/S2.The 96 ferroptosis-related genes(FRGs) in HNSCC were obtained by taking the intersection of...doc]

GCLC

CFTR

LAMP2

GCLM

CD44

AIFM2

HSPA5

CP

ATG5

CASP8

ELAVL1

ACSL4

NEDD4

TFRC

TIGAR

VDAC3

FTL

AURKA

PEBP1

ITGA6

FH

TF

MAPK1

HMOX1

GSS

MAP1LC3A

SLC39A14

HSPB1

CA9

ALOX12

SLC11A2

LPCAT3

STEAP3

EPAS1

NFE2L2

PRDX6

HELLS

GOT1

CISD1

ACSL3

MT1G

BECN1

ATF4

SAT1

SESN2

PRKAA1

ARNTL

HILPDA

MDM2

EGLN1

MYC

RIPK1

YAP1

SLC40A1

SLC39A8

RB1

MAP1LC3B

SAT2

TP53

FANCD2

G3BP1

CDKN2A

SLC7A11

ACSL1

PROM2

ALOX15

PRKAA2

BAP1

ACSL6

CYBB

NGB

VDAC2

GPX4

OTUB1

FTH1

SLC3A2

PCBP1

PRNP

ANO6

ALOX15B

FTMT

SOCS1

MUC1

NF2

HMGB1

PCBP2

ACSL5

MAP3K5

ATG7

MAP1LC3C

PRC1

VDAC1

MIF

NFS1

MAP1LC3B2

NCOA4
